# Optimizing a Nurse-led Transitional Home Visit Program in Preparation for a Randomized Control Trial

**DOI:** 10.1097/pq9.0000000000000012

**Published:** 2017-01-25

**Authors:** Hadley S. Sauers-Ford, Heather Tubbs-Cooley, Angela M. Statile, Rita H. Pickler, Christine M. White, Susan Wade-Murphy, Jennifer M. Gold, Samir S. Shah, Jeffrey M. Simmons

**Affiliations:** From the * Division of Hospital Medicine, Cincinnati Children’s Hospital Medical Center, Cincinnati, Ohio; † Department of Patient Services, Cincinnati Children’s Hospital Medical Center, Cincinnati, Ohio; ‡ Cincinnati Children’s Hospital Medical Center, James M. Anderson Center for Health Systems Excellence, Cincinnati, Ohio; § The Ohio State University, College of Nursing, Columbus, Ohio; ¶ Home Care Services, Cincinnati Children’s Hospital Medical Center, Cincinnati, Ohio; and ‖ Division of Infectious Diseases, Cincinnati Children’s Hospital Medical Center, Cincinnati, Ohio.

## Abstract

**Introduction:**

The Hospital to Home Outcomes study began with the end goal of evaluating the effectiveness of a single, nurse-led transitional home visit (home visit) program, for acutely ill, pediatric patients, which had been piloted at our institution. As part of the overall study design, building on prior randomized control trials that utilized a run-in period prior to the trial, our study team designed an optimization period to test the home visit and study procedures under real-world conditions.

**Methods:**

For this optimization project, there were 3 process improvement goals: to improve the referral process to the home visit, to optimize the home visit content, and to define and operationalize measures of patient- and family-centered outcomes to be used in the subsequent randomized control trial. During the optimization period, a multidisciplinary study team met weekly to review family and stakeholder feedback about the iterative modifications made to the home visit process, content, and outcome measures.

**Results:**

Optimization home visits were completed with 301 families across a variety of discharge diagnoses. The outcomes planned for the clinical trial were tested and refined. Feedback from families and stakeholders indicated that the content changes made to the home visits resulted in increased family knowledge of warning signs to monitor postdischarge. Thirty-one percent of families reported that they altered the care of their child after the home visit.

**Conclusion:**

Through iterative testing, informed by multistakeholder feedback, we leveraged patient and family engagement to maximize the effectiveness and generalizability of the home visit intervention.

## INTRODUCTION

Our institution began piloting a single, nurse-led transitional home visit (home visit) in acutely ill, general pediatric patients in 2012. Initially, the home visit was adapted from a nurse home visit that was neither informed by caregiver reported goals nor systematically tailored to meet individual patient needs.^1^ The Hospital to Home Outcomes (H2O) study began in 2014, with the ultimate goal of evaluating the effectiveness of the home visit through a randomized control trial (trial).^2^ The a priori primary outcomes of the trial included readmission or emergency department revisit within 30 days of an index discharge. In addition, patient- and family-centered outcomes were conceptualized a priori and then augmented during the qualitative phase of the H2O study.^3^ However, before the trial, as the second phase of the overall study design, we optimized the home visit intervention, key study procedures, and patient- and family-centered outcomes using quality improvement methods.

Many clinical trials have included run-in periods before randomization, most commonly to determine how study drugs may affect participants and also to identify participants that may not adhere to the treatment regimen.^4–6^ Researchers then eliminate participants who cannot tolerate or are nonadherent to the study drugs, maximizing the power of the intention-to-treat analysis. Little published work documents optimization of study interventions and processes during these run-in periods.

Clinical trials do not often feature interventions or programs that are developed with patient and other stakeholder involvement. Rather, many interventions tested in trials are developed from prior research or clinical experience of the investigators. By addressing the needs of a wide variety of families during a pretrial optimization period, we believe the home visit intervention, and results from the trial will be more generalizable. This report will describe the optimization phase of the H2O study, which tested and refined a family-centered home visit intervention on acutely ill, general pediatric patients, and revised key study processes, assessed in real-world conditions via stakeholder feedback, to prepare for a randomized control trial.

## METHODS

**Fig. 1.**
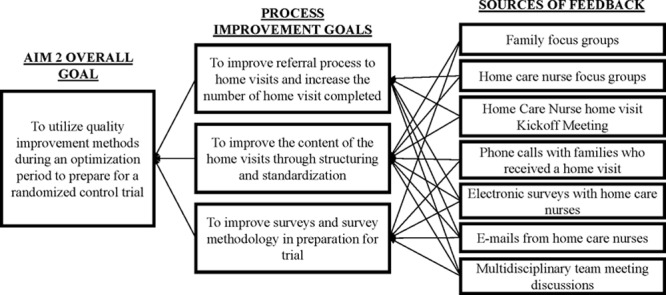
Process improvement goals and feedback sources.

During this optimization period, the multidisciplinary optimization team used quality improvement methods, including tracking quantitative measures and frequent qualitative feedback from key stakeholders, to accomplish 3 sequential improvement goals: (1) to improve the referral process for the home visit; (2) to redesign the home visit content to better address patient and family needs; and (3) to operationalize and refine measures of patient- and family-centered outcomes for the trial (Fig. 1). The optimization team consisted of 3 hospitalists, 2 nurse scientists, 2 members of home care leadership, a project manager, an inpatient nurse, 4 home care nurses, an informatics expert, and 2 clinical research coordinators (study coordinators).

To inform our 3 improvement goals, we reviewed short-term, focused feedback from stakeholders^7^ about the referral process, the home visit, and outcome measures at weekly optimization team meetings. Formal feedback was collected through the following mechanisms: preoptimization focus groups with families and nurses,^3^ follow-up calls with families who received a home visit during the optimization phase, and electronic surveys and emails with home care nurses during optimization (Fig. 1). Modifications to accomplish each improvement goal were discussed within the team; after modifications were implemented, subsequent feedback from families and nurses was reviewed to determine the impact of each modification. Informal feedback was also received and reviewed by study team members throughout the study, and critical process issues were addressed in near real-time, without waiting for weekly meetings.

### Setting and Sample for Optimization

All phases of the H2O study were conducted at a large academic pediatric hospital. Eligible patients were less than 18 years old, with an English-speaking caregiver, not already receiving home nursing visits (such as patients who receive traditional nurse visits for medication infusions), and admitted to either the Hospital Medicine or Neurosciences (Neurology or Neurosurgery) services. These 2 service lines were included due to initial strong interest in the pilot program and to encompass a sample of patients with conditions requiring short-stay hospitalizations, the target sample for the clinical trial. Our Institutional Review Board approved the overall H2O Study, including this optimization period.

### Improving Referral Process

A pilot home visit program had been underway for 18 months when the optimization period began. From August 2012 to March 2014, there was not a standard referral process for home visits; referrals were largely dependent on physician or nurse judgment and memory. Consequently, home visits were underutilized. The team began to improve and standardize the referral process to the visits in April 2014 using quality improvement methods. This process goal was important to ensure that the study team would have enough home visits to test modifications and create a sustainable referral process. Pivotal feedback during this phase came from the weekly team discussions that included the home care nurses scheduling the home visits (Table 1). Goals included increasing the total number of home visits per month and increasing the percent of eligible patients who were referred for a home visit.

**Table 1.**
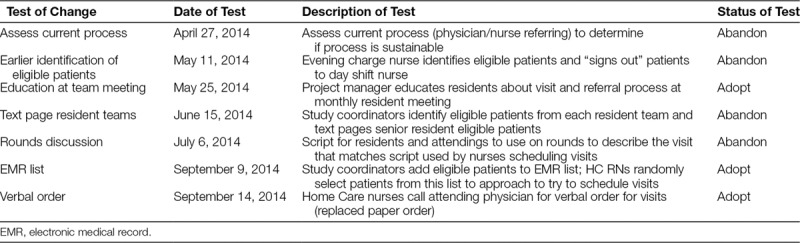
Tests of Change to Improve Home Visit Referrals

### Modifying the Home Visit

Before the optimization period began, the study team evaluated the pilot nurse visit and determined component elements to remove, add, or modify based on the feedback from preoptimization focus groups with 61 families^3^ and from home care nurses (Fig. 1). Once there was sufficient home visit volume to begin testing home visit modifications, the team began to focus on our second improvement goal: redesigning the home visit content. Based on feedback from stakeholders, the study team made 2 key home visit modifications, which provided a standard structure with disease-based customization: the creation of 24 disease-specific templates containing teaching and reinforcement topics for the nurse to review with families, and the creation of “red flag” or warning sign cards to be left with the family. Feedbacks from home care nurses and families about the effects of these key changes were obtained via phone calls and electronic surveys.

During the optimization period, many visits, spanning multiple diagnoses, were completed with families. Open-ended questions to the families who received the visits and the nurses who completed the visits were used to determine if the changes made to the visit resulted in improved qualitative perception of clarity and value of the visit. The parents’ ability to remember the red flags was assessed during the postvisit phone call by comparing the information they remembered to the information they received. We utilized this quantitative measure to try to determine the impact of the red flag cards because improving the understanding of what to look for after discharge was a key conclusion drawn from the preoptimization parent focus groups.^3^ The postvisit phone call questions were modified and adapted as changes were made to the visits to ensure that the questions asked were relevant to the current tests of change.

### Improving Survey Content and Methodology

In addition to obtaining visit feedback, we used a postvisit phone survey to refine and augment measures of patient- and family-centered outcomes for the planned trial. The phone call survey included a validated scale (Post Discharge Coping Scale^8^) to assess the ability of families to cope after hospitalization, as well as a questions created by the study team to assess how the visits worked to provide reassurance to families, and to assess financial implications of the hospital stay. These issues had been key concerns raised during the preoptimization focus groups.^3^ For the Post Discharge Coping Scale, we assessed the variability of item responses to identify floor and ceiling effects using descriptive statistics. Similar to how we generated information to inform visit redesign, we used parent feedback from the calls and multidisciplinary study team meeting discussions to modify survey questions. We also regularly solicited feedback from the study coordinator completing the phone calls to gauge respondent fatigue and question clarity.

## RESULTS

### Improving Referral Process

**Fig. 2.**
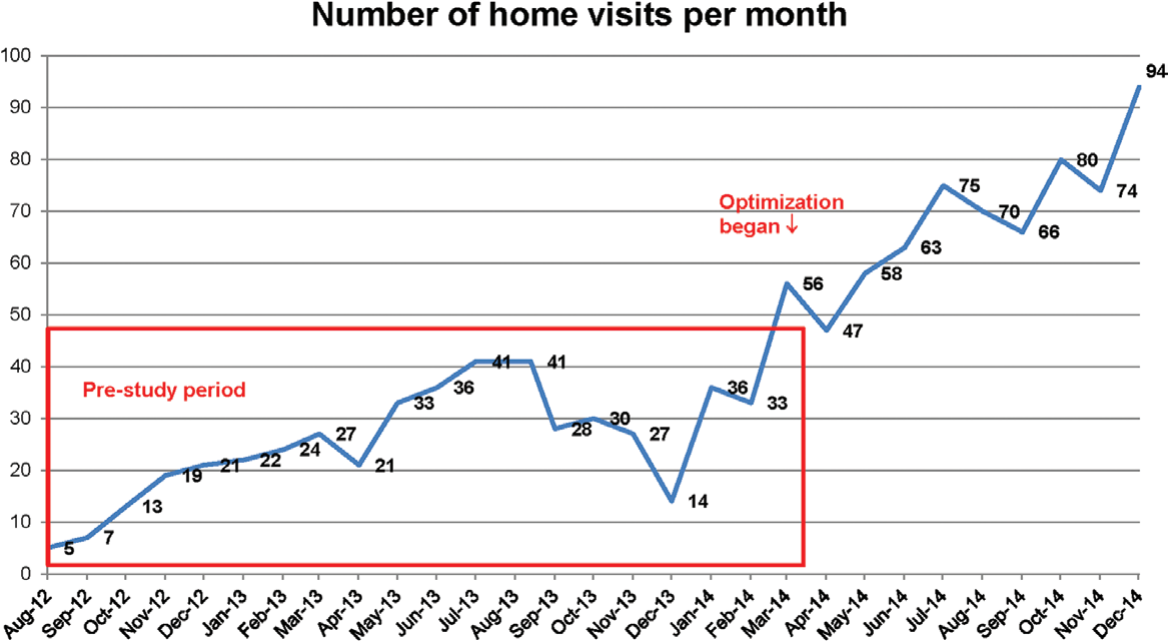
Number of home visits per month.

**Fig. 3.**
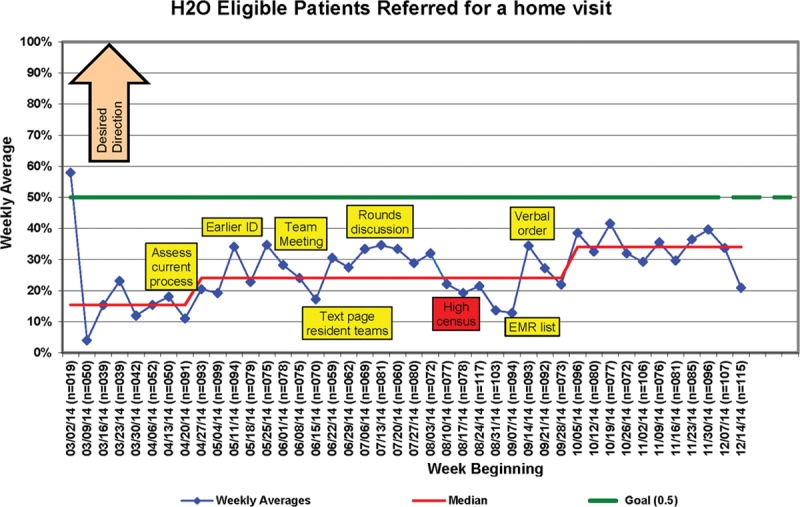
Percent of eligible patients referred for a home visit.

Through modifications to the referral process, the number of home visits completed per month steadily increased (Fig. 2), with over 90 visits completed during the last month of optimization. Initially, referrals were driven by physicians and/or inpatient nurses, so early tests focused on increasing referrals from these groups. However, competing time priorities for these care providers made these processes unreliable. A successful strategy involved the study coordinators creating a daily list from the electronic health record of eligible patients and the home nurses approaching a random selection of these patients before discharge. The median percent of eligible patients referred for a home visit increased from 15.4% to 34% during the optimization period (Fig. 3) though a series of targeted interventions. Table 1 lists the changes tested, some of which were adopted and some of which were abandoned. When the planned clinical trial is complete, home nurses will be able to utilize one idea that was adopted, an automated list to determine patients to approach. Once a patient agreed to the visit, the home care nurses called the attending hospitalist to obtain verbal orders for the visit replacing the previous paper order process, ensuring that each step of the process was performed efficiently by the most appropriate team member, which can also be sustained after the trial.

### Modifying the Home Visits

Visits were completed with 301 families during the optimization period using each type of the disease-specific templates. The most common visits completed were: bronchiolitis (N = 61), asthma (N = 45), pneumonia (N = 29), Apparent Life Threatening Event (N = 22), and cellulitis (N = 20). Postvisit phone calls were completed with 59% (N = 179/301) of families who received a home visit. Calls were primarily completed with mothers (84%) who were White (61%) and single (57%). Call respondents were demographically similar to all patients admitted over the study period. Phone call feedback from families indicated that the changes made to the visit resulted in changes in care and increased knowledge: 31% of families reported that they altered the care of their child after the home visit. One family said, “The nurse made me think about things I hadn’t thought about and I was able to ask questions I forgot to ask, or didn’t know to ask, in the hospital.”

Before the implementation of the red flag cards, 23% of red flags were recalled on the follow-up call; postimplementation, 27% were recalled. Although the proportion of those correctly recalling the red flags did not change significantly, qualitatively, families found the cards beneficial because they served as an easy reference if needed and the cards could be easily shared with other caregivers. Ninety-eight percent of families reported that they felt reassured by the home visit, with one commenting “As it turned out, we did not have a lot of questions. I did not know how the two days between [discharge and the visit] would go so I was happy and felt comforted by the visit.”

### Improving Survey Content and Methods

In addition to providing qualitative feedback about the home visits, the parent phone calls provided data to modify the survey tools for the trial. A number of questions, both those created by the study team and a validated survey tool, were tested. The 11-item Post Discharge Coping Scale^8^ showed variability among respondents; thus, the complete survey will be used in the trial. The study coordinator completing the calls indicated that families seemed less engaged after 15 minutes, so we ensured that the survey did not exceed that time limit by narrowing our questions to those that clearly tied to results from preoptimization focus groups.^3^

Originally, we planned to complete the follow-up phone call 30 days after discharge, but feedback from the study coordinator and our parent study team member suggested that parent recall diminished after 14 days, shortly after return to a normal routine. Thus, we changed the timing of the call to 14 days postdischarge. This decision was solidified after the team formed a partnership with a local health technology firm that will provide us with 30-day reutilization across 19 area hospitals eliminating the need to use the phone call to measure reutilization, a key planned outcome for the trial. Finally, we determined that family preference on call day and time varied widely, so we added a question to the postoptimization trial enrollment survey for the family to identify the best times for calls.

## DISCUSSION

During an optimization period to prepare for a trial, we increased home visit program referrals, redesigned the home visit content, and determined optimal outcome measures and assessment strategies. Further, we engaged many stakeholders through short-term, focused feedback. We believe the resulting optimization through iterative testing informed by multistakeholder feedback leveraged patient and family engagement to maximize the effectiveness and generalizability of the home visit intervention. Because the feedback received came from many families and nurses about home visits spanning many conditions, we believe the home visit is generalizable to a large of variety acute general pediatric and neuroscience discharges. We plan to determine the effectiveness and explore the heterogeneity of the effect of the intervention during the randomized trial because data from the optimization phase are insufficient to draw any conclusions about heterogeneous subgroups.

Using short-term, focused engagement from stakeholders to optimize an intervention for a trial and refine and augment outcomes to study has not been previously described in pediatrics. Other studies, focused on children and adults with chronic conditions have shown success engaging patients and families in research,^9–11^ but engagement of families of children with acute illnesses is infrequent.^12^ Most importantly, in the H2O study, the overall design to discover and test family-centered outcomes beyond reutilization required a method to link the qualitative data generated from focus groups in the first phase to the effectiveness testing in the third phase trial. Within the framework of a mixed-methods study such as ours, an optimization period is a powerful way to capitalize on qualitative insights to improve both the intervention and its evaluation. New insights into outcomes, beyond reutilization, that are important to families were initially framed by focus groups. We subsequently optimized how to measure these outcomes through phone calls with families and other stakeholders. Iterative testing of question clarity, ordering, and timing during the optimization period resulted in a well-tested questionnaire targeted to measure the effect of the home visit on key family-centered issues, such as postdischarge emotional reassurance and out-of-pocket costs associated with transitions.

Although many existing transition interventions in adult or pediatric populations focus on specific disease processes,^13,14^ which are typically chronic, our study will include a broader group of patients with a variety of diagnoses, many of which are acute. Because reutilization is a heterogeneous problem in pediatrics,^15^ our focus on a broad, more generalizable population should produce insights to address this heterogeneity. Our evaluation of changes we made to the visit showed preliminary evidence of improved family knowledge about their child’s acute illness and changes in family care behaviors. Small-scale tests of change allow an intervention to be tested under multiple conditions and modified accordingly, likely improving its broader generalizability.^16^ Our planned trial will provide the definitive test for these knowledge and behavior outcomes and also is powered to reveal any impact on unplanned health care reutilization.

During our efforts to increase referrals to the exiting home visit program, we made iterative changes and used run charts to track progress, methods proven useful in many health care settings, including patient recruitment.^17^ We propose that these methods may also have a role in execution of research, including recruitment and follow-up measurement, augmenting previous work that has used run-in periods focused on effectiveness and efficacy.^4^

An additional interesting finding of our work was that coordination of the home visit process by bedside and home care nurses, as opposed to relying primarily on physicians, was critical to early success. Using an electronic health record–based list allowed the home care nurses to easily identify eligible patients and approach them without waiting for physician referral. After patients expressed interest in receiving a visit, the nurse called the physician for verbal orders, as opposed to waiting for paper orders or for the physician to identify eligible patients. Strong nursing leadership in improving transitions has been shown in adults with chronic conditions.^18–20^ We believe our experiences can be generalized to pediatric transitions, especially for acute disease processes. As transitions continue to play a large part in the global conversation about quality of care, often measured by readmission rates, the involvement of nurses in transitional care referrals and services is essential.

Our optimization results must be considered in the context of several limitations. The population eligible to receive the home visits is limited to patients living within a 55-mile radius from 1 academic pediatric hospital in 1 state, and 2 clinical service lines. Thus, our optimized home visit may not be useful for children with different health conditions or other samples. However, most patients admitted to our institution live within this area and over 300 visits were completed during the study. Moreover, although we only included 2 clinical service lines, the disease processes managed within these services is diverse, with 23 disease-specific templates and 1 general template created to encompass the study population.

Postvisit phone calls were completed only with 59% of the patients who received a home visit during the optimization period. Those who did not participate may have responded differently to the survey questions. However, families of those who participated in the calls were demographically similar to those admitted during the study period. Although multistakeholder feedback informing iterative change is a robust approach to optimization, we did not correlate home visit modifications to a change in outcome, such as readmission, over time, and the gold standard for implementation research. However, our goal for the optimization period was primarily to prepare for the trial, when we will explore the effect of the home visit on key outcomes, including those patient- and family-centered ones refined during the optimization phase.

## CONCLUSION

Through short-term, focused engagement of a variety of stakeholders, including patients, families, and frontline clinical providers, we were able to redesign and refine our home visit intervention and key study processes to prepare for a randomized clinical trial. The changes made to the visit, based on parent and nurse feedback, are expected to result in an improved version of the visit that better addresses patient and family needs in the immediate postdischarge period.

## ACKNOWLEDGMENTS

We would like to thank the H2O Study team for their participation in this work. This work was supported through a Patient-Centered Outcomes Research Institute (PCORI) Award (IHS-1306-0081 to SSS). All statements in this report, including findings and conclusions, are solely those of the authors and do not necessarily represent the views of the Patient-Centered Outcomes Research Institute, its Board of Governors, or Methodology Committee. The Clinical Trial Registry name and number is: Hospital to Home Outcomes, NCT02081846.

## DISCLOSURE

The authors have no financial interest to declare in relation to the content of this article.
